# A Critical View of Current State of Phytotechnologies to Remediate Soils: Still a Promising Tool?

**DOI:** 10.1100/2012/173829

**Published:** 2012-01-04

**Authors:** Héctor M. Conesa, Michael W. H. Evangelou, Brett H. Robinson, Rainer Schulin

**Affiliations:** ^1^Departamento de Ciencia y Tecnología Agraria, Universidad Politécnica de Cartagena, Paseo Alfonso XIII, 48-30203 Cartagena, Spain; ^2^Institute of Terrestrial Ecosystems, Swiss Federal Institute of Technology (ETH Zürich), Universitaestrasse 16, 8092 Zürich, Switzerland; ^3^Agriculture and Life Sciences, Lincoln University, Lincoln, Canterbury 7647, New Zealand

## Abstract

Phytotechnologies are often shown as an emerging tool to remediate contaminated soils. Research in this field has resulted in many important findings relating to plant and soil sciences. However, there have been scant private and public investments and little commercial success with this technology. Here, we investigate the barriers to the adoption of phytotechnologies and determine whether it is still a fertile area for future research. The terminology used in phytotechnologies includes a confusing mish-mash of terms relating to concepts and processes increasing the difficulty of developing a unique commercial image. We argue that the commercial success of phytotechnologies depends on the generation of valuable biomass on contaminated land, rather than a pure remediation technique that may not compare favourably with the costs of inaction or alternative technologies. Valuable biomass includes timber, bioenergy, feedstock for pyrolosis, biofortified products, or ecologically important species.

## 1. Introduction

Soil contamination has become an important environmental problem worldwide because of its detrimental effects on human and ecosystem health, soil productivity, and socioeconomic well-being. In 1994, there were an estimated 22 million ha of contaminated soils worldwide [1]. The European Environment Agency has estimated the total costs for the clean up of contaminated sites in Europe to be between EUR 59 and 109 billion [2]. Soil remediation projects need to incorporate environmental, technical, legislative, and economic factors, all of which are site specific.

Environmental regulations often obligate the remediation of soil if threshold values are exceeded [3–5] or there is an unacceptable risk to agricultural production, ecosystems, or human health [6]. Such regulation has placed an economic imperative to develop low-cost remediation technologies for contaminated soils.

Soil remediation techniques comprise* in situ* (non excavated soil) and *ex situ* techniques (soil is excavated). *Ex situ* remediation can be achieved on site, which requires the presence of a mobile decontamination unit, or off site, which requires that the soil be transported to a treatment facility (e.g., soil washing). Regulators tend to favour *on-site* techniques, which imply soil disposal as close to the source of contamination as possible [5, 7]. The idea of “soil recycling” instead of disposal has been included in official regulations such as the directive 2008/1/EC concerning integrated pollution prevention and control [8]. Moreover, European Union regulators proposed within the same Directive a guideline to select the most suitable technique according to criteria such as environmental friendliness, preexisting scientific knowledge, or required time. Such guidelines leave stakeholders to choose the best remediation technology for their site, considering the economic, environmental, and social variables.

## 2. Biological and Nonbiological Methods

Phytotechnologies have been defined as “the application of science and engineering to study problems and provide solutions involving plants” [9] or as “a set of technologies using plants to remediate or contain contaminants in soil, groundwater, surface water, or sediments” [10].

The costs of conventional methods to remediate soils (Table 1) are comparatively easy to estimate. They are usually based on known production and disposal rates, which permits an accurate estimate of the time required for remediation. However, these conventional engineering techniques can be prohibitively expensive (Table 1). These techniques generally have drawbacks such as generating high amounts of additional wastes that require disposal and are not suited for the treatment of soils that are to be reused for agricultural or similar purposes of plant/biomass production. For example, thermal treatments drastically alter the soil's biological and physical properties, which are the base of soil fertility. Similarly, soil-washing plants produce a residual clay-cake that has to be disposed of in a landfill.

To overcome these problems and to meet regulatory guidelines, scientific research in the last two decades studied *in situ* biological techniques which are environmental friendly as well as cost-effective. Among these techniques, phytotechnologies have received a particular high level of interest from the scientific community.

Phytotechnologies (or phytoremediation) include the removal of pollutants (phytoextraction) [11], their extraction from aqueous solution (e.g., rhizofiltration) [12], transformation (e.g., phytovolatilization) [13], or immobilization (e.g., phytostabilization) [14]. These techniques are frequently shown as promising tools for the remediation of contaminated sites. However, the successful application of these aforementioned technologies in commercial operations or field trials is scarce.

There are many scientific reviews of various aspects of phytotechnology [14–18]. Here, we discuss constraints and opportunities of phytotechnologies in the current environmental market, with a view to elucidating bottlenecks that hinder the commercial uptake of these technologies. We focus on the need to better transfer phytotechnologies to the commercial sector and explore ways to improve the economic viability of these technologies.

## 3. The Need of a Conceptual Normalization

Standardization is a key factor in the development of commercial products and services. For instance, there are standards for the investigation of soil, air, and water contamination under ISO 17020 : 2004 (*General criteria for the operation of various types of bodies performing inspection*) (http://www.iso.org/). Soil remediation does not yet have a standard norm. However, increasing stakeholder demand and commercial needs could bring this about. Conventional remediation techniques, such as soil washing, thermal desorption, soil stabilization plants, would be easier to normalize than phytotechnologies, since yields and physicochemical processes are easier to predict and control than biological parameters, where complex physiological processes and ecological relationships play an overriding role. Therefore, it is imperative that phytotechnologies develop a distinctive image or brand in relation to commercial and environmental issues.

**Figure 1:**
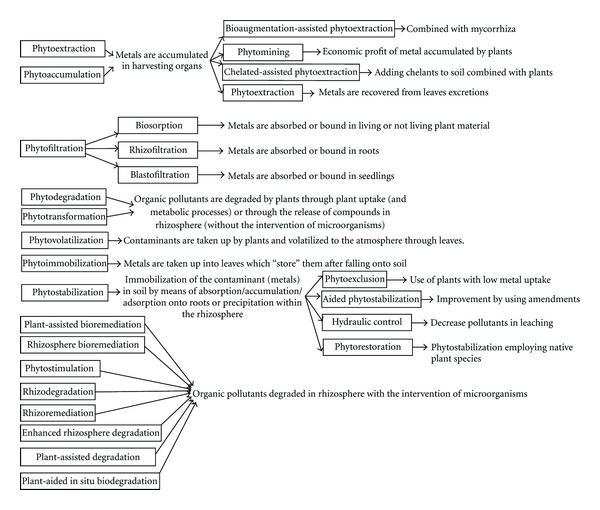
Current classification of most frequently used phytotechnologies for soil remediation.

The number of concepts/processes in phytotechnologies has recently increased with the development of new research domains, and consequently new terms have been introduced to describe new techniques and findings in addition to the renaming of existing techniques (Figure 1). The high number of scientific terms for various processes, mechanisms, or techniques may lead to confusion in the marketplace. The nuances among concepts are often narrow. Non-specialized professionals may have difficulties to elucidate the most suitable technique for a given environmental issue. A review of the common terms that have been used in the last two decades in relation to phytotechnologies shows the lack of normalization among researchers. This is a natural consequence of the scientific progress. However, this confusion may hinder commercial acceptance. In particular, there is a common but wrong belief in the remediation market, as well as among some scientists, that phytotechnologies and in particular phytoremediation is synonymous with phytoextraction, a technology with limited potential application [19]. A comprehensive view of all the terms in use is even difficult for scientists. This can be seen in relation to rhizodegradation, defined as the use of rhizosphere processes involving microorganisms to remediate soils with organic pollutants [20]. That process has also been referred to as phytostimulation, enhanced rhizosphere degradation [20], rhizosphere bioremediation [21], plant-assisted bioremediation, or plant-aided *in situ* biodegradation [22].

In other cases, some terms promote confusion in relation to the mechanisms and biological processes that are involved: phytoimmobilization as it was described by Kaplan et al. [23] included two steps: first, metal phytoextraction and then, sequestration in top soil after leaf fall. Phytostabilization as defined by USEPA [22] refers to the contaminant immobilization in soil by accumulation/adsorption onto roots or precipitation within the rhizosphere, without previous translocation into leaves. The term, phytoextraction, introduced recently by Manousaki et al. [24], refers to the recovery of metals from plants that have the ability to excrete them. This implies phytoextraction, followed by excretion, and then deposition onto top soil, from where they must be removed. When phytostabilization is performed with the goal of returning contaminated land to its former natural state using native plants, the term phytorestoration [25] seems more adequate. Recently, phytoexclusion has been defined as a new technique within phytostabilization [17] to describe the use of excluders, that is, plants that have low bioaccumulation coefficients (shoot/soil metal concentration quotients). Although scientifically justified, the distinction between phytostabilization, phytorestoration, and phytoexclusion is unlikely to be made in the remediation market. Similarly, phytoremoval [26] could be considered as a synonym for phytoextraction. Phytopolishing [27] or plant-assisted remediation [28] are just other forms for phytoremediation.

For Alkorta et al. [20], phytofiltration included rhizofiltration (use of roots) and blastofiltration (the use of seedlings) to adsorb metals from water. Arthur et al. [16] referred to phytofiltration as the use of plant material (living and not living) to recover metals, and to rhizofiltration when roots were employed. Gardea-Torresdey et al. [29] employed the term phytofiltration when referring to the use of plant-derived materials for removing heavy metals from aqueous media.

Phytodegradation [30], which is also called phytotransformation [22], was applied for organic compounds that are degraded, either within the plants or through compounds such as enzymes released into the rhizosphere but without the intervention of microorganisms. Rhizodegradation describes the same process involving microorganisms. If in a second step, the contaminants (organic and elemental such as As, Hg, Se, etc.) are volatilized, then we talk about phytovolatilization.

Phytomanagement [31] describes the engineering or manipulation of soil-plant systems to control pollutant fluxes in the environment. Thus, the goal of phytomanagement may be simultaneously to alleviate deficiencies in essential trace elements such as Zn in produced crops and to reduce environmental risks posed by elevated concentrations of these elements in the soil. A key component of phytomanagement is that it should either cost less than other remediation technologies, or be a profitable operation, by producing valuable plant biomass products. Phytomanagement expresses the aggregation of complex phytoremediation techniques without distinguishing among involved processes.

The commercial success of phytotechnologies requires the distinction of scientific and commercial goals, namely, between processes and techniques. The explosion of new terms may bring an additional problem for the commercial development of phytotechnologies because it may confuse nonspecialized stakeholders who are not familiar with these fields. There is an imperative for researchers to clarify these concepts. Two points are essential: the need of a conceptual redefinition and the elucidation of the most attractive commercial nomenclature. We discuss these issues below.

## 4. Phytotechnologies Must Include Economics: The Need of a Conceptual Change

The first articles detailing phytotechnologies emphasised the low costs of this emerging technology. After more than two decades of scientific development in this topic, recent reviews [18] still consider phytotechnologies as an emerging tool, showing that the reliability of phytoremediation, even inside the scientific community, has not yet been achieved. Thus, it is unsurprising that nonscientific stakeholders in contaminated sites are sceptical about its current applicability or future prospects. Initial estimates of the phytoremediation market by Glass [32] considered it to have a market potential worldwide of 34–54 billion US dollars. Virtually none of this potential has been materialized in the subsequent decade.

**Figure 2:**
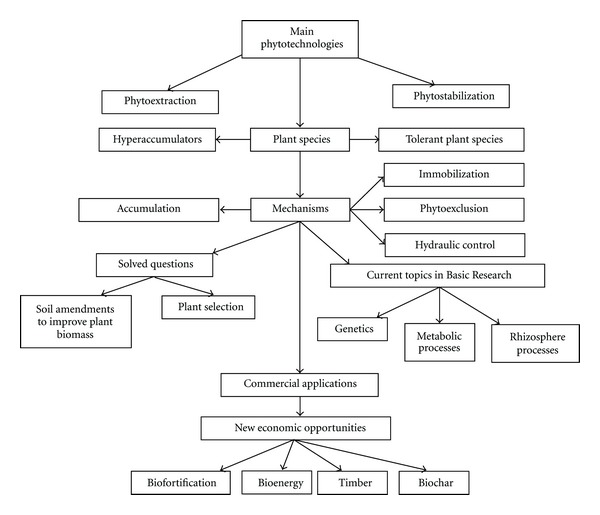
Phytostabilization and phytoextraction application. Current and future development.

Current fundamental research in phytotechnologies (Figure 2) centres on two fields: (1) genetics/physiology/biochemistry in order to increasing plants' tolerance and metabolism of organic pollutants and/or trace elements [33] and (2) rhizosphere processes that influence the phytoavailability of pollutants [28]. Although these topics provide insights into scientific questions, the problem of phytotechnologies low commercial attractiveness due to its lack of revenues still remains. To overcome this issue, it has been proposed to use phytotechnology projects as a way of obtaining profitable outputs (Figure 2)

The original concept of phytoremediation focused on phytoextraction, while phytostabilization received much less attention [13]. The initial focus of phytotechnology was to remove pollutants from soils, by degradation and volatilization in case of organics and extraction in case of metals. Suitable phytoextraction projects relied on high metal extraction rates by the plant species used for remediation [34]. Thus, the research focused on the search for hyperaccumulator plant species [35] or using biotechnology (study of metabolic mechanisms, genetic engineering) to increase metal uptake [15, 36]. This development has resulted in many important findings in plant science that relate to plant-pollutant interactions [37, 38] and to the selection of plant species with hyperaccumulation characteristics [39].

For some common metals, such as Pb, there are no reliable reports of any hyperaccumulator species; therefore, chelant-assisted phytoextraction offered a possible solution. In chelant-assisted phytoextraction, various aminopolycarboxylic acids have been applied to soil to enhance the solubility of trace element cations [40, 41]. Although plant uptake is increased, chelant-assisted phytoextraction has been comprehensively discredited [42, 43], because of the high leaching: plant uptake ratio of the contaminants and the persistence of chelants in the environment.

Successful phytoextraction requires the cleansing of the soil to a level that complies with environmental regulations. Field trials or commercial operations that demonstrate successful phytoextraction are conspicuously absent. Selenium volatilization using genetically engineered *Brassica juncea* (L.) is one of the few examples of a successful field application of phytoextraction [44]. Theoretically, repeated cropping of plants could cleanse contaminated sites, provided the harvested amounts of metals exceed further inputs, until the soil metals concentrations in the long term reach acceptable levels [31].

The development of phytoextraction brought additional issues of practicability, such as the further treatment of biomass, accumulation of pollutants in food chain, or the social and institutional acceptability of using of transgenic plants [45], which were ignored for decades. Back-of-the-envelope calculations show that phytoextraction is not suitable to remediate soils with moderate or high heavy metal contents since it would take an unacceptable time to remove those [31, 46]. Such calculations, for example, rule out the use of phytoextraction in former mining areas. Moreover, there are concerns regarding the entry of metals into the food chain [47]. There has been a progressive shift away from phytoextraction towards phytostabilization. Most plants growing on metalliferous soils are not hyperaccumulators, but excluders of heavy metals. The use of excluders is the base of phytostabilization (Figure 2). Excluder plants may also transform metals into less toxic or mobile forms without extracting them from soil [34] through absorption and accumulation by roots, adsorption onto roots, or precipitation within the rhizosphere [48]. Recent reviews and studies indicate that phytostabilization has more scope of application than phytoextraction [17]. 

The term phytotechnology relates to biochemical processes that can permanently modify an ecosystem. Plants affect evapotranspiration rates, mobilise, immobilise, or extract metals and other chemicals from soil, introduce organic matter into soil, and also release a variety of chemicals by exudation [19]. The original categorization of a project as phytostabilization or phytoextraction may change as the project progresses. Recently, Robinson et al. [31] and Domínguez et al. [49] have extended the phytoremediation concept to more applied projects, showing it as an integrative tool to manage restoration works at large scale using plants for hydraulic control and limitation of metal uptake. This new way of understanding phytotechnologies as phytomanagement is based on the use of the contaminated land for the production of economic yield [31]. Here, remediation is redefined within a dynamic system, which maintains the risks of the contaminants at a safe level and where the factor “soil” generates an economic gain (energy crops, pasture, biofortified products, etc.). That means that phytotechnologies no longer have the sole goal of soil remediation but also of generating economic benefits, and this necessitates redirecting current research lines to more applied aspects. 

### 4.1. Economic Evaluation

**Figure 3:**
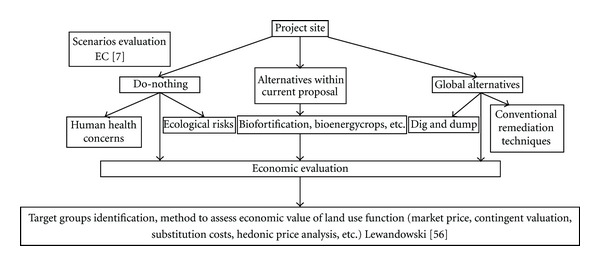
Scheme of evaluation steps in a project remediation site.

According to Lewandowski et al. [50], the quantification of land use functions in biophysical terms requires site-specific information on the landscape, site conditions, or plant species and the identification of target groups which may have benefits from phytotechnologies (farmers, authorities, industries, etc.). This implies that evaluation costs are site-specific and, therefore, that general economic assumptions or yield rates cannot be estimated without site-specific studies. As an initial step in the economic evaluation of phytomanagement projects, some scenarios have to be established in the decision-making process. Potential scenarios (Figure 3) in waste management according to the EC [7] are the following:

- a do-nothing scenario (“business as usual”), without investments,
- some available alternatives inside the current proposal,
- global alternatives to the project.

One of the main barriers for the application of phytotechnologies is the absence of economic studies or cost evaluations. Efforts have been recently made in studying at local or regional scales the economic profits of bioenergy production in contaminated lands [51, 52]. The presentation of phytoremediation as novel low cost remediation technology may not be borne out if the time when the land is out of production is taken into consideration. In a real cost evaluation, “time consuming” is assumed to be an additional cost, and this makes the cost of phytoremediation uncertain because it is difficult to evaluate. Large remediation operations usually come in association with big projects (urban, commercial, industrial). Most commercial soil remediation occurs in relation to the growth of urban areas, where low levels of contaminations must be reached, change of soil use or toxic spills, where urgent solutions are needed to maintain socio-political acceptance. In these cases, conventional remediation options are often the best option due to their rapidity, despite their high initial cost. This makes it difficult for phytotechnologies to compete. Therefore, they are relegated to projects with low economic value and the following profile: (a) long term period is possible; (b) current use of soil does not imply risks for people/ecosystems. These kinds of projects are usually restricted to marginal areas without short term economic value, such as former mining areas [47, 53], landfills, abandoned shooting ranges [54], or postindustrial sites [55].

Lewandowski et al. [56] assessed economic value of phytoremediation combined with biomass production in a Cd polluted soil. According to these authors, assessing the economic value of that combined option requires the accounting of market price of the biomass, contingent valuation/willingness-to-pay or substitution costs/replacement costs.

### 4.2. Commercial Management

There are differences between Europe and North America in the commercial management of phytotechnologies. North America has higher private investment in phytotechnologies, and this has resulted in a larger number of profitable private companies. In contrast, Europe is more focused on solving fundamental issues and in describing biological mechanisms [57]. Consequently, North America is far ahead of Europe in the application and commercialisation of phytotechnologies. Phytoremediation companies have undergone structural and conceptual development in the last 15 years. The first phytoremediation companies were regional manufacturers offering phytoextraction of radionuclides and trace elements (e.g., Phytotech Inc.), or the removal of organics from soil and groundwater by means of trees (e.g., Phytokinetics Inc.). Many of these companies went out of business, because there are few sites that can be remediated solely by phytoextraction. Nowadays, for field-scale remediation, phytoremediation companies team up with large engineering firms, if the latter do not already have an integrated phytoremediation division. Some phytoremediation companies have abandoned the clean up business and practice phytomanagement: most phytotechnology projects aim to prevent contaminant leaching into groundwater (Ecolotree) and the treatment of effluent (Bioplanta). As phytoremediation is a field that is highly dependent on the economic climate, some companies such as Bioplanta Inc. have found additional sources of income, such as methane production in bioreactors or the extraction of active compounds from plants to boost profits. Although some companies have made profits from the field of phytoremediation, the actual breakthrough of this technique has yet not been made. The reason could be that in contrast to other environmental technologies such as renewable energy production by solar panels, wind turbines, and so forth, this technique does not produce profitable outputs. The use of contaminated land for the production of energy crops could reduce the importance of cleanup times while producing a revenue stream [51]. Moreover, “pure environmental” benefits (e.g., CO_2_ friendly, increase of biodiversity) must be considered and, therefore, incorporated to the economic evaluation.

### 4.3. Emerging Opportunities

Biofortification aims to increase the concentration of essential trace elements in crops to improve human health and agricultural productivity [58]. Micronutrients such as Fe and Zn are deficient in many diets [59]. Physiologically accumulated micronutrients in plants provide a more readily assimilable source of micronutrients than in the form of inorganic supplements [58]. Some field experiments have shown positive results in relation to Se-fortified vegetables [60]. According to Qaim et al. [61], biofortification is likely to gain in importance in the future, as indicated by the large number of related international research programs recently launched. However, more evidence, including medical trials, toxicity assessment, and appropriate dosages is needed before biofortified products be available for the consumers [62, 63].

Phytotechnologies may increase revenues from nonproductive polluted soils if linked with biomass production [56, 64]. Among the different options, the production of biofuels, nonconsumable agricultural products, or wood is economically viable in many countries. Biofuels are highly depended on subsidies, and they will establish themselves commercially only when the oil price exceeds 120 US $ per barrel [65]. In contrast to biofuel production, production of biochar could give a new perspective on the production of biomass on contaminated land. Biochar is charcoal created from the pyrolysis of biomass. The addition of biochar to soil has been suggested as a means to sequester carbon, thereby reducing the effects of human-induced climate change caused by CO_2_ emissions [66].

The economics of biochar will depend on the plant species used and the farming intensity applied (fertilisers, pesticides, herbicides) as this will affect operational costs. Monocultures are vulnerable under inadequate soils or stress conditions (drought, pathogens) making it an important issue to reach sustainable system to guarantee the economic and ecological stability of the local environment [67]. Therefore, crops are best when rotated or, if possible, a multispecies community must be established. Additionally, the use of food producing from agricultural species such as maize, rape, and sunflower contains the risk that the contaminants contained in the harvest may enter food or fodder, thus posing a risk to humans and animals. In addition to agricultural species, tree plantation could be an option, with rotation periods of up to 25 years [68]. This rotation would be similar to rotation periods of forestry and would have reduced expenses compared to short-term rotations because of lower plantation costs. This implementation, however, would make it unsuitable for short-term financial outputs. Since the land is anyway not producing revenue and the trees have phytostabilising potential, short rotation periods are less important.

Another possibility may be phytomining; the use of plants to mine metals [69] has been shown to be economically feasible for certain metals such as nickel [70, 71]. However, practical aspects, especially efficiency of land use will prohibit its widespread use [31]. Greater efforts in developing rates, modelling, treatment times, and monitoring schemes are still necessary to provide a better practical view of this technology [30, 72].

### 4.4. Adoption of a New Concept

Traditionally, phytotechnology projects had the sole aim of remediating the site. There is a lot of information on candidate plant species for these technologies, especially regarding plant metal-accumulation and tolerance [46, 73]. Mesocosm experiments have been employed to reveal the capabilities and limitations of soil conditioners [74]. The results of some successful field trials in specific sites are also available [31, 44].

Unlike other remediation systems such as capping and soil removal, phytotechnologies systems are site dependent. It is impractical to conduct long-term field trials to optimise phytotechnology systems for each site. Therefore, models that calculate the performance of phytotechnology systems are crucial. Such models could eliminate unnecessary field trials by revealing where phytotechnologies will likely meet environmental regulations and where they are more cost-effective than competing technologies. Without such models, the improvement of phytotechnologies requires field trials to determine the feasibility and optimal management strategies for the site. This further delays the operation.

**Figure 4:**
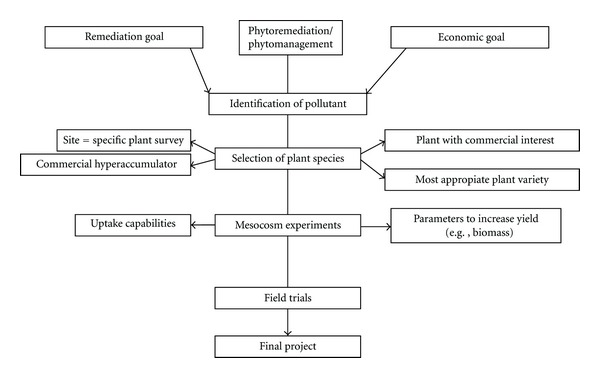
Phytoremediation decision tree.

Approaching phytotechnologies from a commercial perspective, such as has occurred in the United States, is more likely to increase the attractiveness of this technology.  Figure 4 shows the current implementation pathway for phytotechnologies using this system. The critical success factor for this system is that the remediation goal and the economic goal are given equal weightings.

## 5. Conclusions

Research in phytotechnologies has enhanced our understanding in the fields of plant and soil sciences. However, more effective and commercially feasible techniques are still required. Therefore, to make phytotechnologies more commercially attractive, we propose:

- clearly distinguishing processes from techniques to improve communication and cooperation by the commercial sector,
- the exploitation of new economic opportunities such as the production bioenergy, biochar, and biofortified crops,
- the application of economic studies and economic evaluations as well as a new implementation protocol. Phytotechnologies will forever remain a promising tool for soil cleaning if they are not linked to the production of valuable biomass products.

## Figures and Tables

**Table 1:** Current prices and yields from conventional soil remediation techniques in Europe (prices from 2008 to 2009, own data).

	Soil desorption	Soil washing	Soil stabilisation	Soil oxidation	Dig and dump
Cost (euro/t)	40–100	25–40	40–50	60–70	60–90
Yield (t/h)	25–50	35–80	~20	~50	—
